# Ending homelessness among people with mental illness: the At Home/Chez Soi randomized trial of a Housing First intervention in Toronto

**DOI:** 10.1186/1471-2458-12-787

**Published:** 2012-09-14

**Authors:** Stephen W Hwang, Vicky Stergiopoulos, Patricia O’Campo, Agnes Gozdzik

**Affiliations:** 1 Centre for Research on Inner City Health, The Keenan Research Centre in the Li Ka Shing Knowledge Institute of St. Michael’s Hospital, 30 Bond Street, Toronto, Ontario, M5B 1W8, Canada; 2 Division of General Internal Medicine, Department of Medicine, University of Toronto, Toronto, Ontario, Canada; 3 Department of Psychiatry, University of Toronto, 250 College Street, 8th floor, Toronto, Ontario, M5T 1R8, Canada; 4 Dalla Lana School of Public Health, University of Toronto, Health Sciences Building, 6th floor, 155 College Street, Toronto, Ontario, M5T 3M7, Canada

## Abstract

**Background:**

The At Home/Chez Soi (AH/CS) Project is a randomized controlled trial of a Housing First intervention to meet the needs of homeless individuals with mental illness in five cities across Canada. The objectives of this paper are to examine the approach to participant recruitment and community engagement at the Toronto site of the AH/CS Project, and to describe the baseline demographics of participants in Toronto.

**Methods:**

Homeless individuals (n = 575) with either high needs (n = 197) or moderate needs (n = 378) for mental health support were recruited through service providers in the city of Toronto. Participants were randomized to Housing First interventions or Treatment as Usual (control) groups. Housing First interventions were offered at two different mental health service delivery levels: Assertive Community Treatment for high needs participants and Intensive Case Management for moderate needs participants. Demographic data were collected via quantitative questionnaires at baseline interviews.

**Results:**

The effectiveness of the recruitment strategy was influenced by a carefully designed referral system, targeted recruitment of specific groups, and an extensive network of pre-existing services. Community members, potential participants, service providers, and other stakeholders were engaged through active outreach and information sessions. Challenges related to the need for different sectors to work together were resolved through team building strategies. Randomization produced similar demographic, mental health, cognitive and functional impairment characteristics in the intervention and control groups for both the high needs and moderate needs groups. The majority of participants were male (69%), aged >40 years (53%), single/never married (69%), without dependent children (71%), born in Canada (54%), and non-white (64%). Many participants had substance dependence (38%), psychotic disorder (37%), major depressive episode (36%), alcohol dependence (29%), post-traumatic stress disorder (PTSD) (23%), and mood disorder with psychotic features (21%). More than two-thirds of the participants (65%) indicated some level of suicidality.

**Conclusions:**

Recruitment at the Toronto site of AH/CS project produced a sample of participants that reflects the diverse demographics of the target population. This study will provide much needed data on how to best address the issue of homelessness and mental illness in Canada.

## Background

Homelessness continues to be a serious health and social problem in large urban areas in Canada. In 2009, there were approximately 498 shelters serving homeless individuals and families nationwide with a total of 17,256 beds, of which 31% were in the province of Ontario [1,2]. In Toronto (population 2.5 million), more than 5,000 people are homeless on any given night [3,4]. Of this number, more than three quarters (79%) live in shelters, 8% on the street, 6% in correctional facilities, 4% in health care or treatment facilities and another 3% in Violence Against Women shelters [5]. In 2008, approximately 28,000 unique individuals used homeless shelters in Toronto over the course of the year [6].

The prevalence of physical and mental illness and addictions are significantly higher among people who are homeless than in the general population [7]. Studies suggest that between one-quarter to one-third of people experiencing homelessness are affected by schizophrenia, major depressive disorder, or bipolar affective disorder [8,9]. Substance abuse is consistently a strong predictor of housing loss and is also the most common co-morbidity among individuals who have severe mental illness [10-15].

In Toronto, mental illness is highly prevalent among people experiencing homelessness. A 1998 study using diagnostic interviews concluded that approximately two-thirds (66%) of homeless people in Toronto had a lifetime diagnosis of mental illness, a prevalence rate two to three times higher than what is observed in the general population [16]. A 2007 survey of homeless individuals in Toronto found that 35% self-reported a previous diagnosis of mental illness, with the most common diagnoses reported being depression (17%), anxiety (11%), bipolar affective disorder (8%), schizophrenia (5%) and post-traumatic stress disorder (5%) [17]. The 2009 Toronto Street Needs Assessment, a comprehensive census and survey of homeless people across the city, found that 24% of all respondents indicated that they needed mental health support in order to help them achieve housing [5].

The problem of homelessness is Toronto is made more complex than in many other Canadian urban areas due to the diversity of the population and the large numbers of recent immigrants. Almost half (47%) of Toronto residents belong to ethno-racial groups [18]. Half of all Toronto residents are immigrants to Canada, and almost 20% are recent immigrants who arrived between 1996 and 2006 [18]. About one third of homeless people in Toronto are immigrants to Canada, and these individuals experience particular barriers to accessing services related to race, language, and social stigma [19,20].

### Homelessness interventions

Two distinct intervention models for addressing the needs of homeless individuals with mental illness have been recognized in recent years. The first, often called a Treatment First model, follows a step by step progression that begins with outreach, followed by treatment, and eventually leads to the ultimate goal of permanent housing [21]. Treatment First programs focus on providing individuals with services to first address mental illness and substance abuse, while the ultimate goal of accessing housing can only be reached once the person has achieved adequate stability [22]. The second, more recently developed intervention model, known as Housing First, views housing as a human right, and focuses on providing safe and affordable housing that is not contingent on acceptance of psychiatric treatment or abstinence from alcohol or drugs [23]. The Pathways to Housing program was the originator of a Housing First consumer orientated program in New York City during the 1990’s [15,21]. Clients of Housing First are offered a choice of subsidized scattered-site apartments, and intensive mental health supports are typically provided through a multidisciplinary Assertive Community Treatment team. In the Housing First model, the main goal is to provide the client with housing, within an environment of consumer choice and harm reduction [15,21-23].

Studies have shown that the Housing First model can result in positive housing and health outcomes for participants and for society in general [15,21,24-35]. However, the Housing First model has only been systematically assessed by a few randomized controlled studies, and there is little evidence to date regarding the effectiveness, cost-effectiveness and critical elements of this model within a Canadian context [28,36-38]. Both health care and social policy differences exist between the United States and Canada, and while the Housing First approach has shown to be an effective intervention in the United States, it is not clear if the model will be equally effective in Canada. In addition, several gaps remain in our knowledge regarding the effectiveness of the Housing First model. First, it is not known if the Housing First approach will be equally effective among different subpopulations (including individuals of different age, sex, those who are Aboriginal or belong to other racialized ethnicities, immigrants, or those with concurrent disorders). Second, to date, no cost-benefit analysis or cost effectiveness analysis has been completed that compares the Housing First model to standard care. Third, there has been a lack of fidelity assessment among Housing First programs; these could be used to determine if elements of the approach have been implemented and how they relate to key outcomes. Fourth, an Intensive Case Management-based Housing First has been proposed as a less costly alternative for meeting the needs of homeless individuals with moderate mental health needs; this approach has not been evaluated for effectiveness compared to standard care, and will be evaluated as part of the At Home/Chez Soi project.

### The At Home/Chez Soi (AH/CS) project

The At Home/Chez Soi (AH/CS) project is the largest randomized controlled trial of the Housing First intervention worldwide. AH/CS is a 4-year multi-site (Toronto, Moncton, Montreal, Winnipeg and Vancouver) randomized controlled trial of the effectiveness of a Housing First intervention funded by the Mental Health Commission of Canada (MHCC). Study participants are provided with housing free of the requirements of sobriety or treatment in addition to access to a wide array of health and social services. Consumer choice is central to the project and treatment is not required for participants to continue living in their housing. The ultimate goal of this project is to assess the effectiveness and cost-effectiveness of the Housing First approach in the Canadian context, and to be to better able to advise policy and programs for homeless individuals in Canada.

The findings from the Toronto site of the AH/CS project, in particular, will provide knowledge on how to best implement a Housing First approach in other large service-rich urban centres worldwide. Because of the ethnic diversity of the Toronto homeless population, the findings from this site will also better inform the effectiveness of the Housing First model in various subpopulations.

### Objectives

The purpose of this paper is to describe the unique context of the Toronto site of the AH/CS project, and provide descriptive data on study participants at baseline. This paper also highlights some of the key challenges experienced during the implementation of the research at the Toronto site, with a focus on 1) recruitment strategies and challenges, 2) community engagement and 3) relationship building with the pre-existing network of services.

## Methods

### Context of the Toronto AH/CS site

As part of the national multi-site study, the Toronto site shares an overall study design in common with all AH/CS study sites (see [36] for details). However, the context and development of the project in Toronto features a number of unique elements. In terms of urban context, the Toronto site differs from many other cities in that there is a relatively large array of existing services available for individuals experiencing homelessness. These services include drop-in centers, emergency shelters, meal programs, street outreach services, and supportive and alternative housing. Toronto has a large network of mental health services that serve both housed and homeless individuals. These services include inpatient and outpatient services, case management, assertive community treatment, court support services, crisis programs, and ethno-racial focused agencies. In addition, the City of Toronto operates the Streets to Homes program, which engages with homeless individuals living outdoors using a modified Housing First approach without any rent subsidies [24].

Another unique aspect of the Toronto site project development is the involvement of People with Lived Experience (PWLE) of homelessness and mental health problems. The Toronto site engaged with a group of PWLE as part of the ongoing planning, development, and execution of the study. PWLE group members provided advice and expertise at a series of meetings that were held during the study development process. This group has continued to advise on all aspects of the project, including service provision and research protocols throughout the duration of the study.

The Toronto site has also developed a unique intervention for the AH/CS study that provides ethno-racial intensive case management (ER-ICM) for participants with moderate needs who belong to a racialized group. High rates of mental health problems have been observed in immigrants, refugees and ethno-racial individuals in Canada and worldwide [39-45]. However, reports from Canada, US, UK and Australia suggest that immigrant and ethno-racial groups use mental health services less frequently compared to non-immigrants and experience significant barriers to care [39,46-50]. The unique Toronto-based ER-ICM intervention aims to address the unique challenges faced by this group.

Finally, the Toronto site of AH/CS is being run by an inter-sectoral partnership to promote integration across the mental health, housing, social services, and research sectors. This partnership involves the City of Toronto’s Shelter, Support and Housing Administration Division (SSHA), St. Michael’s Hospital’s Centre for Research on Inner City Health (CRICH), three community-based mental health services organization (Across Boundaries, COTA Health and Toronto North Support Services), and a housing team (which includes SSHA and Housing Connections, a subsidiary of the Toronto Community Housing Corporation, which offers social housing solutions for people looking for affordable housing in Toronto). The project governance chart is provided in Additional file 1: Figure 2.

### Recruitment strategy

**Figure 1:**
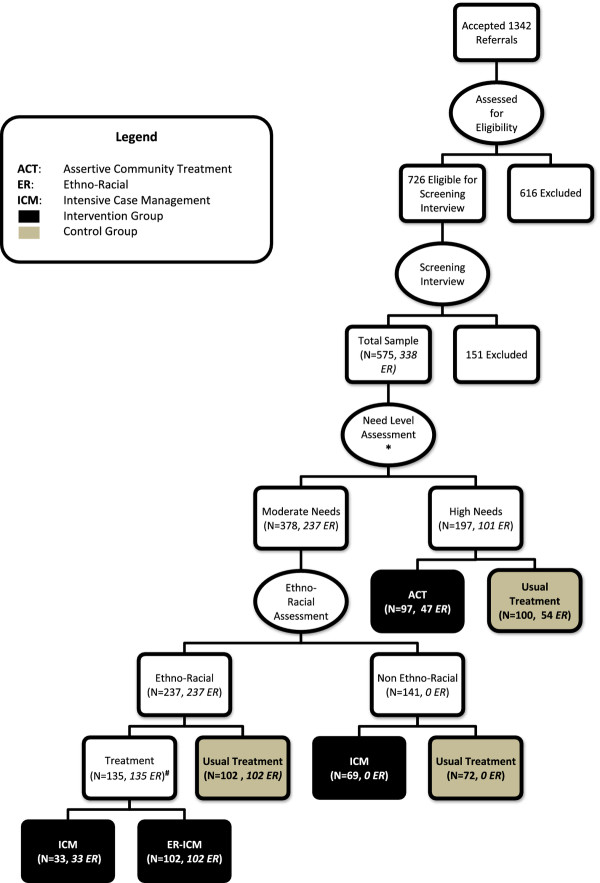
**Participant flow through study.** *Operational definitions for High Needs vs. Moderate Needs. High Needs participants must have: 1) A score <62 on the Multnomah Community Ability Scale (MCAS); AND 2) A Mini International Neuropsychiatric Interview (MINI) diagnosis of current psychotic or bipolar disorder OR observations of psychotic disorder by referral source; AND 3) One of: **a**) Two or more hospitalizations for mental illness in any one year in last five years OR **b**) Co-morbid substance use OR **c**) Recent arrest or incarceration (or don't know or declined to answer). All other eligible participants were considered Moderate Needs. ^**#**^Participants with Moderate Needs who self-identified membership in an Ethno-Racial group were given a choice to participate in a regular ICM program or an Ethno-racial focused ICM program, as long as space was available in both groups.

The target population for this study were homeless adults with serious mental illness residing in the Toronto area. The goal was to recruit 560 participants, stratified based on their level of service need and ethno-racial group membership (see Figure 1 for study flow chart). Eligibility criteria for the AH/CS study have been described in detail elsewhere (see [36]) and are summarized in Table 1. The study was approved by the Research Ethics Board of St. Michael’s Hospital in Toronto, and was registered with the International Standard Randomized Control Trial Number Register (ISRCTN42520374).

**Table 1:** **Study eligibility (A) and ineligibility criteria (B) and reasons (and numbers) for exclusion of referral (C**)

			
**A. Eligibility criteria**		**B. Exclusion criteria**	
1. ≥18 years old		1. Relatively homeless^3^	
2. Absolutely homeless^1^ or Precariously housed^2^		2. Illegal status (not a Canadian citizen, landed immigrant, refugee or refugee claimant)	
3. Presence of serious mental disorder with or without co-existing substance use problem (no formal diagnosis necessary for study entry)		3. No serious mental disorder	
4. Not currently receiving Assertive Community Treatment (ACT) or Intensive Case Management (ICM)		4. Current client of an ACT or ICM	
**C. Excluded referrals and causes**			
**Excluded prior to screening interview (N = 616)**	***N***	**Excluded post screening interview (N = 151)**	***N***
Referrals delayedprior to potential screening*	445	Did not meet serious mental disorder criteria	59
Not absolutely homeless/precariously housed	44	Declined consent	54
Current client of ACT/ICM	29	Was not absolutely homeless/ precariously housed	16
Did not show up for Screening interview	24	Unable to provide informed consent	4
Lost contact with study staff	17	Lack of space in relevant group	5
Did not meet serious mental disorder criteria	14	Current client of ACT/ICM	9
Did not meet requirements of ICM	9	Incomplete referral	1
No longer interested in participating in study	9	Participant wasn’t aware of referral and declined participation	1
Found housing	4	Withdrew	1
Not eligible to receive social assistance income support	4		
Not informed of study by referral source	3		
Moved to another country	2		
In justice system	2		
Deceased	2		
Deported	2		
Secured own housing	1		
Withdrew	1		
Unable to provide consent	1		
Declined permission to be referred	1		
Not of age	1		
Too sick	1		

*Referrals were kept for a period of up to three months. During the early phases of the study there were more referrals than staff available to accept new participants; therefore, many referrals were delayed beyond the three month period and were excluded prior to screening.

^1^**Absolutely homeless:** no fixed place to stay for at least the past 7 nights with little likelihood of finding a place in the upcoming month.

^2^**Precariously housed:** housed in single room occupancy (SRO), rooming house, or hotel/motel as a primary residence AND in the past year have a history of 2 or more episodes of being Absolutely Homeless OR one episode of being absolutely homeless of at least 4 weeks duration in the past year.

^**3**^**Relatively homeless:** individuals who inhabit spaces that do not meet the basic health and safety standards, such as living in overcrowded or hazardous conditions.

Any organization or individual was able to refer potential participants to the intake coordinator for assessment of eligibility for the study. The study sought to obtain the majority of referrals from Toronto’s extensive network of service providers for homeless and mentally ill individuals. A core target group of more than 80 shelters, drop-in centres, hospitals, outreach programs, mental health services, and community health centers were encouraged to refer potential participants. Individuals were allowed to self-refer to the study, but, whenever possible, they were asked to identify a service provider who could refer them.

A brief referral form that summarized eligibility criteria was distributed to the referral network, as well as being posted online. Information gathered on this form included contact information for the referral source, the potential participant’s current living situation and contact information, symptoms or service use history that might indicate the presence of a mental illness, and the reasons why the individual might require Intensive Case Management or Assertive Community Treatment. The study intake coordinator used this information to screen each referral and determine whether the individual met the criteria for study eligibility. In many cases, the intake coordinator would obtain additional information to determine eligibility. If eligibility criteria were met, a meeting with the potential participant was arranged for further screening and to obtain informed consent.

Targeted recruitment was undertaken to ensure appropriate representation of homeless individuals from specific demographic groups and living situations. Recruitment targets were based on the characteristics of the overall homeless population of Toronto, as determined by a comprehensive 2006 census of homeless people [3]. Using these data, targeted recruitment was undertaken to ensure that approximately 25% of participants were women and approximately 40% of participants were from immigrant and ethno-racial groups. Based on homeless census data, an additional goal was to recruit about 75% of participants at shelters, 17% from people living on the street, and 8% at health care facilities, prisons, and jails.

### Study eligibility and establishment of need level

In order to meet study inclusion criteria, participants had to 1) be 18 years of age or older; 2) be either absolutely homelessness or precariously housed (for definitions see Table 1); and 3) have a serious mental disorder with or without a co-existing substance use disorder. Participants were excluded if they 1) were current clients of another Assertive Community Treatment or Intensive Case Management program; 2) did not have legal status in Canada as a citizen, landed immigrant, refugee, or refugee claimant; 3) were relatively homeless (for definition see Table 1).

**Table 2:** Characteristics and details of intervention groups

**Needs level**	**Moderate needs**		**High needs**
**Treatment acronym**	**ICM**	**ER-ICM**	**ACT**
Treatment name	Intensive Case Management	Ethno-Racial Intensive Case Management	Assertive Case Management
Rent allowance^#^	$600	$600	$600
Service team	Intensive case management (ICM)	Intensive case management (ICM) with focus on ethno-racial diversity	Assertive community treatment (ACT)
Name of service team	Toronto North Support Services	Across Boundaries	COTA Health
Participant/staff ratio	20:1	20:1	10:1
Availability to participant	5 days/week; 8 hours/day	7 days/week; 12 hours/day	7 days/week; 24 hours/day
Location of service	In the community	In the community	In the community
Services provided	▪ Participants are matched to a Case Manager who works with the participant to develop a service plan	▪ Same as the regular ICM services with the addition of services oriented towards the ethno-racial population	▪ Participants have access to entire ACT team, which will include a psychiatrist and nurse
	▪ Focus is on independent living and providing supports that increase personal independence over time	▪ This service provider takes a more holistic approach to mental health care that recognizes spiritual, emotion, mental, physical, social, economic, cultural, linguistic and broader environmental aspects of life including social determinants of health	▪ ACT team provides all of the relevant services, including case management, initial/ongoing assessment, psychiatric services, employment and housing assistance, family support and education, substance use services and other services and support to allow the individual to live successfully in the community
	▪ Case manger will accompanies participant to appointments (with psychiatrist/social workers, etc.)	▪ Main goal of model is to assist participants to help build support network, including with family and friends	
Additional services		▪ Programs and initiatives that are available to participants:	
		– integrative peer support	
		– skills building	
		– social and recreational activities	
		– support groups, alternative and complementary therapies (including art and music therapy)	
		– creative expressions	
		– community kitchen and	
		– individual and community outreach	
Crisis support services	Yes	Yes	Yes
Non-English services^1^	No	Yes	No
Length of treatment	Minimum 1 year	Minimum 1 year	Minimum 1 year

^1^Services are provided in languages other than English.

The presence of a serious mental disorder was established at the time of screening for study entry and was defined by diagnosis and disability based on several lines of evidence: 1) behavioural observations made by referral sources; 2) indicators of functional impairment; 3) history of recent psychiatric treatment; and 4) current presence of an eligible diagnosis, as identified by the DSM-IV criteria in the Mini International Neuropsychiatric Interview 6.0 (MINI). Participants with a documented prior diagnosis or a MINI diagnosis made at study entry of at least one of the following Axis I diagnoses were considered eligible for this study: 1) major depressive episode; 2) manic or hypomanic episode; 3) mood disorder with psychotic features; 4) panic disorder; 5) post-traumatic stress disorder; and 6) psychotic disorder [36].

### Randomization

The AH/CS study is a randomized controlled trial (RCT) that follows participants for 24 months. The randomization procedures have been described in detail elsewhere [36]. Prior to randomization, all eligible participants were stratified based on the extent of disability and severity of psychiatric problems into “high needs” or “moderate needs” for mental health services groups, based on an algorithm described elsewhere and summarized in Figure 1[36]. The criteria for establishing need level included community functioning, mental disorder diagnosis, co-morbid conditions, prior hospitalizations and incarcerations, as well as results from the MINI and the Multnomah Community Ability Scale (MCAS) (see Measures below). In order to be considered “high needs”, participants had to have a MCAS score of less than 62 ***and*** have a MINI diagnosis of psychotic or bipolar disorder or an observation of psychotic disorder on the eligibility screening instrument (i.e., answered “yes” to at least two of Questions 6–10, see Additional file 2: Table 5) ***and*** had to meet one of following three criteria: 1) had indicated “yes” (or “don’t know” or declined) to having been hospitalized for mental illness two or more times in any one year in the last five years; ***or*** 2) have indicated co-morbid substance use; ***or*** 3) have answered “yes” (or “don’t know” or declined) to recent arrest or incarcerations. All other participants who met study eligibility criteria but did not meet the criteria for the “high needs” group were considered “moderate needs” [36].

Specific to the Toronto site, moderate needs participants were further stratified by ethno-racial group membership prior to randomization. Participants who did not self-identify as belonging to an ethno-racial group were randomized to either Housing First with Intensive Case Management (ICM) or a Treatment as Usual (TAU) control group. Participants who indicated membership in an ethno-racial group were allowed to choose between assignment to the regular ICM intervention or a Housing First with ethno-racial specific ICM intervention (ER-ICM). Choice was allowed as long as there was available space in both intervention arms. As a result, ethno-racial participants were assigned to both the regular ICM and ER-ICM intervention groups (Figure 1).

Participants identified as High Needs were randomized to either Housing First with Assertive Community Treatment (ACT) or TAU, regardless of ethno-racial group membership status. Since there was no unique ethno-racial intervention for participants with high needs, both non-ethno racial and ethno-racial high needs participants randomized to receive treatment were provided services by the same Assertive Case Management team (see Figure 1).

### Measures

#### MINI international neuropsychiatric interview 6.0 (MINI 6.0)

The MINI 6.0 is a short, structured diagnostic interview used for psychiatric evaluation, typically administered in approximately 15 minutes. In the AH/CS study, we focused specifically on the following modules of the MINI 6.0: 1) major depressive episodes; 2) manic or hypomanic episodes; 3) post-traumatic stress disorder; 4) panic disorder; 5) mood disorder with psychotic features; 6) psychotic disorder; 7) alcohol dependence; 8) alcohol abuse; 9) substance dependence; 10) substance abuse; and 11) suicidality. Diagnosis of at least one of the first six disorders listed met the eligibility criteria for the presence of a serious mental disorder [36].

The MINI has been validated against several much longer diagnostic interviews, including the Structure Clinical Interview for DSM Diagnoses (SCID-P), and against the Composite International Diagnostic Interview for ICD-10 (CIDI) [51,52]. In comparison to both the SCIP-P and ICD-10, the MINI has shown good or very good concordance and high sensitivity for most diagnoses, with a high level of reliability [51,52].

#### Multnomah community integration scale (MCAS)

The MCAS is a 17-item instrument that measures the degree of functional ability of adult clients who have severe and persistent mental disorders and who live in the community [53]. Items are rated on a 5-point scale and are grouped into four categories; 1) interference with functioning 2) adjustment to living; 3) social skills; and 4) behavioural problems The MCAS has good internal consistency (Cronbach’s α=0.90) with high test-retest reliability (ICC = 0.83 for total scale) [53]. Anchor and interview probes were developed by Dickerson *et al.*, and increased test-retest reliability (ICC = 0.96 for total scale) [54].

Barker *et al.* (1994) proposed criterion scores for interpreting levels of disability in individuals with severe mental illness: total MCAS scores of 17 to 47 indicate severe disability, 48 to 62 indicate moderate disability and 63 to 85 indicate mild disability [53]. Other investigators report MCAS ratings in the 40s for inpatients [55], in the 50s for ambulatory patients receiving a high level of community support [56], and in the 60s for clients in lower intensity outpatient care [55].

Other data collected as part of the AH/CS project, including additional survey elements and qualitative data elements, are described in further detail elsewhere [36].

### Intervention groups

The AH/CS project follows a Housing First intervention model. A detailed description of the project intervention groups has been published elsewhere [36]. All participants randomized to the intervention groups receive a rental allowance of $600 per month (until the end of the study funding period in March 2013). The rent allowance is paid directly to the landlord; however, the participants are named on the lease and entitled to all rights and obligations as a tenant under provincial legislation. The study budget also includes an allowance for furnishing and moving costs. Housing is provided in scattered site private market apartments.

Study participants in the intervention groups additionally receive support services, which differ depending on their service needs and ethno-racial group membership (see Table 2 for details of services provided in each intervention group). Services are offered throughout the study, but client participation in treatment for their mental health and addiction problems is voluntary (i.e. tenancy is not tied to participation in treatment). Participants are not required to abstain from drugs or alcohol. The only requirements of the intervention group participants are that rental payments from their income are made directly to landlords and that participants meet with a project case manager at least once a week.

### Treatment as usual group

Participants randomized to the TAU control group are able to access a variety of pre-existing programs and services in the city of Toronto. TAU participants were provided with information about the availability of such services in the community and were directed to both mainstream and homeless-specific health services for care.

### Data collection and analysis

All study participants completed a series of quantitative questionnaires during the screening and baseline interviews and are participating in follow-up interviews every three months up to a maximum follow-up duration of 24 months. These surveys inquire about the participants’ housing situation, physical and mental health status, substance use and overall quality of life [17,32,33,51,52,54,57-94]. Table 3 outlines the tools used and their subject matter, including those specifically designed for use at the Toronto site. Descriptive statistics were calculated for each of the collected baseline quantitative measures. T-tests and chi-square tests, as appropriate, were used to test for significant differences between the treatment and control participants, stratified by need level, at baseline.

**Table 3:** Instruments used in At Home/Chez Soi Toronto site study at screening and baseline visits, their acronyms and references

**Acronym**	**Full name**	**References**
ACC	Health Service Access Items	[17,57,58]
CIS	Community Integration Scale	[59-61]
CMC	Comorbid Conditions List	[33,57,62]
CSI	(Modified) Colorado Symptom Index	[32,63-65]
DHHS	Demographics, Housing, Vocational and service Use History	[66-68]
EQ-5D	EuroQuol 5D	[69-71]
FS	Social Support Items and Food Security	[72,73]
GAIN-SPS	Global Assessment of Individual Need – Substance Problem Scale	[74,75]
HSJSU	Health, Social Justice Service Use Inventory	[76-82]
III	Interviewer Impressions Items	
MCAS	Multnomah Community Ability Scale	[54,83,84]
MINI	MINI International Neuropsychiatric Interview	[51,52,85,86]
QoLI-20	Quality of Life Index – 20 Item	[87-89]
RAS-22	Recovery Assessment Scale – 22 items	[90-92]
SCNR	Eligibility Screening Instrument	
SF-12	SF12 Health Survey v. 2	[71,93,94]
T-SU	Substance Use	
T-DIET	Diet	
T-PH	Preventative Health	
T-PHYS	Physiological Measures	
T-SB	Supplementary BL	
T-CD-RISC-2	Resilience	
T-PSC	Stress	

## Results

### Participant recruitment

Between October 2009 and June 2011, a total of 1,342 referrals were received. The largest proportion of referrals was received from shelter services (41%) but hospitals (14%), drop-in centres (12%), outreach programs (10%), mental health services (8%) were also key sources of recruitment. A total of 726 (54%) referrals passed the initial review for eligibility and underwent further screening. Reasons for exclusion, prior to and after the screening interview are provided in Table 1.

A total of 575 individuals (43% of all referrals) met all eligibility requirements, provided informed consent, and completed screening and baseline interviews. Of this number, 197 were stratified to the high needs group and 378 to the moderate needs group. Randomization resulted in the assignment of 301 individuals to one of the three intervention groups and 274 individuals to a control group. Among the intervention groups, 97 were assigned to the ACT intervention group, 102 to the ICM intervention group and 102 to the ER-ICM intervention group (see Figure 1).

### Baseline sample characteristics

Table 4 summarizes the baseline characteristics of participants for the sample overall and stratified by intervention group and need level group. Among study participants, the most common age group was 40–49 years old (32%), most were male (69%), born in Canada (54%), single/never married (69%), without dependent children (71%), and absolutely homeless (93%). Within each need level group, intervention and control participants did not differ in any baseline characteristics except in two instances where marginally significant differences between the intervention and control participants were noted, both within the moderate needs group: the prevalence of current alcohol dependence was higher in control participants (33%) compared to intervention participants (24%, p = 0.046), and the number of participants who provided information suggesting a history of a learning disability was higher in control (41%) compared to intervention participants (31%, p = 0.044). Although this study specifically sought to recruit homeless individuals with mental illness, a large proportion of participants (70%) reported no written documentation of a mental health diagnosis. However, the prevalence of mental health conditions and concurrent disorders was high as determined by the MINI [52]. The prevalence of mental health conditions in our sample at baseline was as follows: suicidality (65%), substance dependence (38%), psychotic disorder (37%), major depression (36%), alcohol dependence (29%), post-traumatic stress disorder (PTSD) (23%), mood disorder with psychotic features (21%), panic disorder (14%), alcohol abuse (14%), mania/hypomania (11%) and substance abuse (9%).

**Table 4:** Baseline characteristics of study participants by need level and randomization group

	**Total sample**^**1**^**(N=575)**		**Moderate needs**^**2**^**(N=378)**			**High needs**^**2**^**(N=197)**		
			**Control (N=174)**	**Treatment (N=204)**		**Control (N=100)**	**Treatment (N=97)**	
	**Mean ± SD or N (%)**	**Missing N (% of 575)**		**Mean ± SD or N (%) **	***P value***		**Mean ± SD or N (%)**	***P value***
**Demgraphics**								
Age		0 (0.0)			*0.861*			*0.340*
<30	138 (24.0)		41 (23.6)	51 (25.0)		19 (19.0)	27 (27.8)	
30-39	134 (23.3)		41 (23.6)	47 (23.0)		22 (22.0)	24 (24.7)	
40-49	183 (31.8)		53 (30.5)	67 (32.8)		34 (34.0)	29 (29.9)	
≥50	120 (20.9)		39 (22.4)	39 (19.1)		25 (25.0)	17 (17.5)	
Gender		0 (0.0)			*0.059*			*0.075*
Female	170 (29.6)		54 (31.0)	65 (31.9)		19 (19.0)	32 (33.0)	
Male	394 (68.5)		113 (64.9)	138 (67.6)		79 (79.0)	64 (66.0)	
Other^3^	11 (1.9)		7 (4.0)	1 (0.5)		2 (2.0)	1 (1.0)	
Country of birth		1 (0.2)			*0.668*			*0.610*
Canada	312 (54.3)		90 (51.7)	101 (49.5)		60 (60.0)	61 (63.5)	
Other	262 (45.6)		84 (48.3)	103 (50.5)		40 (40.0)	35 (36.5)	
Native language		0 (0.0)			*0.301*			*0.145*
English	369 (64.2)		104 (59.8)	123 (60.3)		69 (69.0)	73 (75.3)	
French	17 (3.0)		3 (1.7)	9 (4.4)		1 (1.0)	4 (4.1)	
Other	189 (32.9)		67 (38.5)	72 (35.3)		30 (30.0)	20 (20.6)	
Ethnic or cultural identity		0 (0.0)			*0.265*			*0.361*
Aboriginal	28 (4.9)		8 (4.6)	10 (4.9)		3 (3.0)	7 (7.2)	
Ethno-racial^4^	338 (58.8)		102 (58.6)	135 (66.2)		54 (54.0)	47 (48.5)	
Non ethno-racial^5^	209 (36.3)		64 (36.8)	59 (28.9)		43 (43.0)	43 (44.3)	
Marital status		3 (0.5)			*0.943*			*0.650*
Divorced/separated/widowed	152 (26.4)		51 (29.3)	57 (28.1)		25 (25.0)	19 (20.0)	
Married/cohabitating with partner	21 (3.7)		6 (3.4)	8 (3.9)		4 (4.0)	3 (3.2)	
Single, never married	399 (69.4)		117 (67.2)	138 (68.0)		71 (71.0)	73 (76.8)	
Number of children		8 (1.4)			*0.260*			*0.105*
0	410 (71.3)		125 (72.7)	139 (68.1)		74 (77.1)	72 (75.8)	
1	80 (13.9)		23 (13.4)	38 (18.6)		8 (8.3)	11 (11.6)	
2	55 (9.6)		18 (10.5)	15 (7.4)		14 (14.6)	8 (8.4)	
≥3	22 (3.8)		6 (3.5)	12 (5.9)		0 (0.0)	4 (4.2)	
**Homelessness**								
Current status		0 (0.0)			*0.633*			*0.284*
Absolutely homeless	535 (93.0)		161 (92.5)	186 (91.2)		97 (97.0)	91 (93.8)	
Precariously housed	40 (7.0)		13 (7.5)	18 (8.8)		3 (3.0)	6 (6.2)	*0.207*
Total length of homelessness During lifetime (years)	5.25 ± 6.19	13 (2.3)	4.86 ± 5.82	4.56 ± 5.63	*0.599*	7.13 ± 7.34	5.57 ± 6.42	*0.122*
Longest period of homelessness (years)	2.92 ± 4.54	8 (1.4)	2.58 ± 3.61	2.50 ± 4.12	*0.855*	4.07 ± 5.81	3.29 ± 5.27	*0.388*
**Education and employment**								
Education history		2 (0.3)			*0.346*			*0.273*
< High school	279 (48.5)		75 (43.4)	103 (50.5)		53 (53.5)	48 (49.5)	
Completed high school	108 (18.8)		36 (20.8)	34 (16.7)		22 (22.2)	16 (16.5)	
Some post-secondary school	186 (32.3)		62 (35.8)	67 (32.8)		24 (24.2)	33 (34.0)	
Employment status		2 (0.3)			*0.924*			*0.975*
Unemployed	549 (95.5)		165 (94.8)	193 (94.6)		97 (98.0)	94 (97.9)	
Employed	24 (4.2)		9 (5.2)	11 (5.4)		2 (2.0)	2 (2.1)	
**Mental health**								
MCAS		0 (0.0)			*0.224*			*0.931*
More disability (17-47)	29 (5.0)		0 (0.0)	1 (0.5)		14 (14.0)	14 (14.4)	
Some disability (48-62)	192 (33.4)		14 (8.0)	9 (4.4)		86 (86.0)	83 (85.6)	
Mild disability (63-85)	354 (61.6)		160 (92.0)	194 (95.1)		0 (0.0)	0 (0.0)	
Documented health record of a mental disorder	161 (28.0)	9 (1.6)	36 (20.7)	44 (22.1)	*0.739*	44 (44.4)	37 (39.4)	*0.475*
MINI Results		0 (0.0)						
Current depressive episode	206 (35.8)		79 (45.4)	92 (45.1)	*0.953*	18 (18.0)	17 (17.5)	*0.931*
Current manic Episode or hypomanic episode	61 (10.6)		16 (9.2)	25 (12.3)	*0.340*	7 (7.0)	13 (13.4)	*0.137*
Current PTSD	134 (23.3)		48 (27.6)	61 (29.9)	*0.620*	11 (11.0)	14 (14.4)	*0.469*
Current panic disorder	81 (14.1)		34 (19.5)	38 (18.6)	*0.822*	3 (3.0)	6 (6.2)	*0.284*
Current mood disorder with psychotic features	119 (20.7)		33 (19.0)	38 (18.6)	*0.933*	25 (25.0)	23 (23.7)	*0.833*
Current psychotic disorder	215 (37.4)		44 (25.3)	55 (27.0)	*0.712*	60 (60.0)	56 (57.7)	*0.746*
Current alcohol dependence	166 (28.9)		57 (32.8)	48 (23.5)	*0.046*	30 (30.0)	31 (32.0)	*0.766*
Current substance dependence	218 (37.9)		69 (39.7)	74 (36.3)	*0.499*	32 (32.0)	43 (44.3)	*0.075*
Current alcohol abuse	80 (13.9)		17 (9.8)	30 (14.7)	*0.147*	16 (16.0)	17 (17.5)	*0.774*
Current substance abuse	52 (9.0)		13 (7.5)	19 (9.3)	*0.521*	8 (8.0)	12 (12.4)	*0.310*
Current suicidality					*0.127*			*0.589*
*Low*	203 (35.3)		64 (36.8)	73 (35.8)		38 (38.0)	28 (28.9)	
*Moderate*	112 (19.5)		47 (27.0)	37 (18.1)		13 (13.0)	15 (15.5)	
*High*	60 (10.4)		15 (8.6)	25 (12.3)		9 (9.0)	11 (11.3)	
**Functional and cognitive impairment**								
Cognitive impairment								
Was in special class in school	170 (29.6)	18 (3.1)	48 (28.1)	58 (29.3)	*0.796*	30 (30.6)	34 (37.8)	*0.300*
Received extra help with learning in school	196 (34.1)	18 (3.1)	63 (36.8)	62 (31.3)	*0.263*	33 (34.0)	38 (41.8)	*0.274*
Thinks that has learning problem/disability	207 (36.0)	22 (3.8)	70 (41.4)	62 (31.3)	*0.044*	37 (39.4)	38 (41.3)	*0.787*
Was told have learning problem/disability	180 (31.3)	19 (3.3)	56 (33.3)	57 (28.6)	*0.332*	33 (34.4)	34 (36.6)	*0.754*
Functional Impairment								
Needs assistance to meet nutritional needs	101 (17.6)	1 (0.2)	21 (12.1)	30 (14.8)	*0.443*	20 (20.0)	30 (30.9)	*0.078*
Needs assistance to maintain adequate personal hygiene	129 (22.4)	0 (0.0)	23 (13.2)	25 (12.3)	*0.779*	42 (42.0)	39 (40.2)	*0.798*
Needs assistance/unwilling to access needed resources	413 (71.8)	1 (0.2)	110 (63.2)	141 (69.5)	*0.200*	79 (79.0)	83 (85.6)	*0.228*
Needs assistance to manage finances	425 (73.9)	2 (0.3)	126 (72.4)	136 (67.0)	*0.255*	79 (79.8)	84 (86.6)	*0.203*
Needs assistance to acquire and maintain social network	455 (79.1)	0 (0.0)	127 (73.0)	163 (79.9)	*0.113*	85 (85.0)	80 (82.5)	*0.631*
**Referral sources**		0 (0.0)			*0.309*			*0.112*
Community health centres	5 (1.0)		2 (1.1)	1(0.5)		2 (2.0)	0 (0.0)	
CMHA (Canadian Mental Health Association)	31 (5.4)		10 (5.7)	7 (3.4)		5 (5.0)	9 (9.3)	
Drop-ins	67 (11.7)		21 (12.1)	26 (12.7)		12 (12.0)	8 (8.2)	
Hospitals	80 (13.9)		12 (6.9)	21 (10.3)		25 (25.0)	22 (22.7)	
Social supports programs and justice systems^6^	25 (4.3)		6 (3.4)	11 (5.4)		2 (2.0)	6 (6.2)	
Mental health services	45 (7.8)		10 (5.7)	24 (11.8)		8 (8.0)	3 (3.1)	
Other^7^	27 (4.7)		9 (5.2)	12 (5.9)		5 (5.0)	1 (1.0)	
Outreach programs	57 (9.9)		15 (8.6)	17 (8.3)		9 (9.0)	16 (16.5)	
Shelters	238 (41.4)		89 (51.1)	85 (41.7)		32 (32.0)	32 (33.0)	

^1^ For the total sample, percentages shown were calculated as proportion of the total sample (N = 575) and therefore the column totals for each variables will not add up to 100% if data was missing (see adjacent column with N and % Missing in total sample).

^2^ For calculation of the moderate needs and high needs group values, percentages are calculated out of the total **available** data (excluding missing), therefore column totals for each variables add up to 100%. However, questions with “yes/no” answers, only the proportion of individuals who indicated “yes” are provided)

^3^ “Other” category includes individuals who identify as Transgendered, Transsexual or Other.

^4^ “Ethno-racial” includes participants who indicated the following ethnicities: Black (includes Black-Africa, Black-Caribbean, and Black-Canada), East Asian, Indian-Caribbean, Latin American, Middle Eastern, South Asian, South-East Asian and Mixed Ethnicity.

^5^ The “Non Ethno-Racial” category includes participants who indicated the following ethnicities: White-Canada, White-Europe and Other.

^6^ This category includes Ontario Works and Ontario Disability Support Program (both provincial social support programs) as well as various justice system referrals (including jails, bail programs, and probation and parole offices).

^7^ The “Other” category includes the City of Toronto Assessment and Referral Centre (ARC), Aboriginal Legal Services, hospitals outside of the city of Toronto, self-referrals and any other agencies who did not clearly fit into any of the other categories.

## Discussion

### Baseline characteristics

Randomization at the Toronto site of the At Home/Chez Soi randomized controlled trial of Housing First was successful and resulted in no significant differences between intervention and control groups in key baseline demographic and exposure variables. In terms of demographics, our study population resembles that of other studies of homeless individuals with mental illness in large urban centers; the sample is comprised predominantly of single males, many of whom have a history of alcohol and substance abuse [95-97]. A 1997 study reported that Toronto shelter users (n = 300) were relative young (mean age = 33.4 ± 11.4 years), mostly male (78%) and non-Caucasian (28%), of whom a majority had a lifetime history of mental illness (67%) and/or substance abuse or dependence (68%) [95]. A recent larger study (n = 1189) of homeless shelter user similarly found that most were single/never married (63%), male (54%), white (56%), with many having recent mental health (37%) and alcohol (29%) problems [96].

The service needs algorithm designed for this study succeeded in classifying participants into a higher needs group and a moderate needs group. Participants in the high needs group had experienced longer periods of homelessness compared to those with moderate needs and had a higher degree of functional impairment. Our classification algorithm identified high needs participants using multiple criteria that included major psychiatric symptoms, substance abuse, criminal justice involvement, and community functioning. These criteria are often associated with individuals who are chronically homeless [98-100]. Chronically homeless individuals comprise a small proportion of the overall homeless population (estimates range between 10 to 22%), but they suffer from a disproportionately high level of disability and have been shown to be the most intense users of health care and social services [98-100].

### Recruitment strategies: challenges and successes

Recruitment of marginalized and hard-to-reach groups for research purposes is always difficult, and this study was not an exception. Recruitment was aided, however, by a pre-existing network of mental health and homelessness services in Toronto. Although all recruitment was implemented by the AH/CS site research team, the referral system was guided by initial consultation with a Referrals Working Group, which met monthly during the early stages of the project and included the research team and representatives of most homeless service sectors in Toronto. The Working Group was invaluable in providing feedback and advice to ensure community engagement, designing recruitment protocols that would be acceptable to homeless people and frontline social service providers, and problem-solving around recruitment challenges. The Working Group also oversaw the targeted recruitment strategies and ensured that participants reflected the makeup of homeless people in Toronto.

Challenges to recruitment arose in the early days of the study. There were delays in building a research team with the needed training, educating potential referral sources about the study and eligibility criteria, and developing recruitment protocols acceptable to all. This resulted in an unexpectedly low rate of intake for service providers early in the study and the need to negotiate the flow of referrals to meet both recruitment timelines and service provider needs. However, this wait time also allowed for extensive training of the service team and research staff, which allowed for a more smooth recruitment process once the research study was initiated. Later on, as the research team quickly increased recruitment rates in an effort to reach target numbers, the service team’s capacity to intake participants became a limiting factor. Intake visits typically required two case managers, which put additional pressure on teams that were already trying to cope with the unexpectedly high needs of the study population.

The referral-based recruitment process was an important component of the successful recruitment strategy at the Toronto site of the AH/CS study. Most homeless individuals in Toronto already had at least some connection to a service provider at the time the study was initiated. This connection was very helpful because participants had the support of a service provider during the enrolment process, and their ongoing support was particularly important to participants who were randomized to treatment as usual. The referral-based approach also enabled the research team to obtain collateral information about potential participants’ level of function and types of support needed, which was critical to correctly assign people to high or moderate needs groups. The referral-based approach was also important in allowing the targeted recruitment of participants from racialized groups.

### Engaging the community and service providers

During initial engagement with the community, many stakeholders showed an interest and willingness to participate in the project. At the Toronto site, engagement of the community took place through several educational sessions that were either open to the general community or tailored for specific service sectors (e.g., shelter providers, drop-in centers). These sessions provided information on the details of the project, including goals, expectations, the types of participants that would be recruited, and the types of individuals who were and were not appropriate for referral to the study.

However, some community members and service providers initially felt ambivalent towards the project. In particular, there was substantial opposition to the randomization of participants to the treatment as usual group and a fear of letting participants down at the conclusion of project intervention in 2013. Active outreach to service providers that were potential referral sources and ongoing dialogue were important components of the community engagement strategy and helped to address some of these concerns.

The engagement of PWLE of homelessness and mental health problems was a key element of this approach. As early as November 2008, PWLE were included in the initial consultative meetings to introduce the project to the community at large. The PWLE established a community-based group, composed of 22 individuals and representatives from community agencies. The PWLE group began to function as a working committee that meets monthly. PWLE group members are represented on all of the Toronto AH/CS projects’ decision making bodies, including all working groups, the Site Operations Team and the Local Advisory Committee.

Many study participants experienced disappointment during the process of the study, particularly those who were randomized to the TAU group. Efforts were made to ensure that existing support services in Toronto were available and accessible for these individuals, either through their referral sources or research team members. Because the referral sources often had close relationships with the participants, they were able to provide support after the randomization process took place, and were often able to suggest or offer other available services to the participants external to the services provided through the study. Additionally, research staff explained the important role the participant’s contribution would have in increasing knowledge to help other homeless individuals.

## Conclusions

The participants recruited for the Toronto site of the AH/CS project appear to represent an appropriate group for the evaluation of a Housing First intervention for people who experience homelessness and mental illness. Targeted recruitment strategies ensured that the sample was representative of the ethno-racial diversity of Toronto and the characteristics of its homeless population. Findings from the Toronto site of AC/HS will provide policy makers and services providers with important data on the effectiveness of a highly promising intervention to meet the needs of diverse populations experiencing homelessness and mental illness, particularly in large service-rich urban centres worldwide.

## Competing interests

SH, VS, PO, and AG have no competing interests.

## Authors' contributions

SH, VS, PO contributed to the writing of the manuscript and revised drafts of the manuscript. AG analyzed and interpreted the data and drafted the manuscript. All authors read and approved the final manuscript.

## Pre-publication history

The pre-publication history for this paper can be accessed here:

http://www.biomedcentral.com/1471-2458/12/787/prepub

## Supplementary Material

Additional file 1 **Figure 2.** Governance Structure at the Toronto Site of the At Home/Chez Soi Project. Click here for file

Additional file 2 **Table 5.** Screener Questionnaire Questions 6–10. Click here for file
