# Supplementary material for: Ending homelessness among people with mental illness: the At Home/Chez Soi randomized trial of a Housing First intervention in Toronto
Source: BMC Public Health. 2012 Sep 14;12:787. doi: 10.1186/1471-2458-12-787 (PMC3538556; doi:10.1186/1471-2458-12-787)
Supplement: Additional file 1 — Figure 2. Governance Structure at the Toronto Site of the At Home/Chez Soi Project. [file 1471-2458-12-787-S1.docx]

**Figure 2 Governance Structure at the Toronto Site of the At Home/Chez Soi Project**
