# Supplementary material for: Ending homelessness among people with mental illness: the At Home/Chez Soi randomized trial of a Housing First intervention in Toronto
Source: BMC Public Health. 2012 Sep 14;12:787. doi: 10.1186/1471-2458-12-787 (PMC3538556; doi:10.1186/1471-2458-12-787)
Supplement: Additional file 2 — Table 5. Screener Questionnaire Questions 6–10. [file 1471-2458-12-787-S2.doc]

Table 5 Screener Questionnaire Questions 6-10

| **Question Number** | **Question Content** |
| --- | --- |
| 6 | Verbal expression of clearly false or bizarre ideas such as reporting references to self on TV or in newspaper or claiming to be someone he/she clearly is not. |
| 7 | Appears to or reports hearing sounds of people talking when no one is around, or sees visions others do not see. |
| 8 | Extremely difficult to understand because odd use of words, rambling, and/or sudden change of topics. |
| 9 | Indications of extreme sadness together with severe apathy or withdrawal. |
| 10 | Engages in self harm activities such as slashing and/or frequent overdosing. |
